# Systems analysis of *cis*-regulatory motifs in C_4_ photosynthesis genes using maize and rice leaf transcriptomic data during a process of de-etiolation

**DOI:** 10.1093/jxb/erw275

**Published:** 2016-07-19

**Authors:** Jiajia Xu, Andrea Bräutigam, Andreas P. M. Weber, Xin-Guang Zhu

**Affiliations:** ^1^CAS Key Laboratory of Computational Biology and State Key Laboratory for Hybrid Rice, CAS-MPG Partner Institute for Computational Biology, Shanghai Institutes for Biological Sciences, Chinese Academy of Sciences, Shanghai 200031, China; ^2^Institute of Plant Biochemistry, Cluster of Excellence on Plant Sciences (CEPLAS), Heinrich-Heine University, 40225 Düsseldorf, Germany; ^3^Network Analysis and Modeling, IPK Gatersleben, Correnstrasse 3, D-06466 Stadt Seeland, Germany

**Keywords:** C_4_ photosynthesis, cell specificity, *cis* element, evolution, etiolation, systems biology.

## Abstract

Identification of potential *cis*-regulatory motifs controlling the development of C_4_ photosynthesis is a major focus of current research. In this study, we used time-series RNA-seq data collected from etiolated maize and rice leaf tissues sampled during a de-etiolation process to systematically characterize the expression patterns of C_4_-related genes and to further identify potential *cis* elements in five different genomic regions (i.e. promoter, 5′UTR, 3′UTR, intron, and coding sequence) of C_4_ orthologous genes. The results demonstrate that although most of the C_4_ genes show similar expression patterns, a number of them, including chloroplast dicarboxylate transporter 1, aspartate aminotransferase, and triose phosphate transporter, show shifted expression patterns compared with their C_3_ counterparts. A number of conserved short DNA motifs between maize C_4_ genes and their rice orthologous genes were identified not only in the promoter, 5′UTR, 3′UTR, and coding sequences, but also in the introns of core C_4_ genes. We also identified *cis*-regulatory motifs that exist in maize C_4_ genes and also in genes showing similar expression patterns as maize C_4_ genes but that do not exist in rice C_3_ orthologs, suggesting a possible recruitment of pre-existing *cis*-elements from genes unrelated to C_4_ photosynthesis into C_4_ photosynthesis genes during C_4_ evolution.

## Highlight

Time-series RNA-seq data collected from etiolated maize and rice leaf tissues sampled during the greening process were used to identify potential *cis*-regulatory motifs that might be recruited into C_4_ genes from non-photosynthetic genes.

## Introduction

Many of the world’s most productive crop species, such as maize, sorghum, and miscanthus, use C_4_ photosynthesis (Brown, 1999). C_4_ photosynthesis has independently evolved from C_3_ photosynthesis in more than 66 lineages (Sage *et al.*, 2011). Compared with C_3_ photosynthesis, C_4_ photosynthesis has higher water-, nitrogen-, and light-use efficiencies (Zhu *et al.*, 2008; Sage and Zhu, 2011). This higher photosynthetic efficiency is achieved by concentrating CO_2_ at the site of RuBisCO, thereby minimizing the rate of photorespiration (Leegood, 2002). These modifications probably required the evolution of new regulatory mechanisms, in the form of either *cis*- or *trans*-regulatory factors or elements (Sheen, 1999; Hibberd and Covshoff, 2010; Kajala *et al.*, 2012; Griffiths *et al.*, 2013; Aubry, *et al.*, 2014). Elucidation of these regulatory mechanisms underlying cell-specific expression of C_4_-related genes is a major focus of current C_4_ photosynthesis research.

Cell-specific expression of C_4_-related proteins and enzymes is governed by multiple layers of regulation (see reviews by Hibberd and Covshoff, 2010, and Williams *et al.*, 2012). A number of *cis*-regulatory motifs controlling C_4_-specific expression have been identified (Hibberd and Covshoff, 2010). Both 5′UTR and 3′UTR regions can potentially be involved in mediating the cell-specific accumulation of C_4_-related genes (Marshall *et al.*, 1997; Ali and Taylor 2001; Lai *et al.* 2002; Patel *et al.* 2004, 2006; Kajala *et al.*, 2012; Williams *et al.*, 2016). The bundle sheath-specific expression of both *NAD-ME1* and *NAD-ME2* genes is controlled by a segment of the coding sequence in *NAD-ME* and *NADP-ME* subtype species (Brown *et al.*, 2011). Therefore, the regulatory motifs related to cell specificity of C_4_ genes might reside in all segments of the gene, i.e. promoter, coding sequence, 5′UTR, 3′UTR, and intron. Furthermore, many of these *cis*-regulatory elements have been reported to be recruited from pre-existing elements, such as the special coding segment in *NAD-ME* (Brown *et al.*, 2011) and the regulatory elements in the UTR regions of *CA* and *PPDK* (Kajala *et al.*, 2012).

*Cis*-regulatory elements controlling the spatially specific expression patterns of C_4_ genes have mainly been discovered using experimental approaches on the single-gene level, e.g. through deletion analysis (see reviews by Sheen, 1999; Hibberd and Covshoff, 2010). Recent progress in sequencing technology and computational approaches now offers an alternative method to identify candidate *cis*-regulatory motifs involved in the regulation of genes. Several methods have been developed for *cis*-element identification. These methods can be categorized into three major classes: (1) methods based on position weight matrix (PWM), e.g. TRAP (Roider *et al.*, 2007), MATCH (Kel *et al.*, 2003), and SIGNAL SCAN (Prestridge, 1996); (2) phylogenetic footprinting methods, e.g. FootPrinter (Blanchette, 2003), Phyloscan (Palumbo and Newberg, 2010), and PHYME (Sinha *et al.*, 2004); and (3) standard motif-finding algorithms, e.g. Gibbs sampling (Jia and Li, 2012), MEME (Bailey *et al.*, 2006), AlignACE (Roth, 1998), YMF (Sinha, 2003), and Weeder (Pavesi *et al.*, 2006). It is a common practice to combine different approaches to increase the reliability and decrease the false discovery rates of the prediction.

In this study, we aimed to establish a basic routine to identify potential motifs that were recruited during C_4_ evolution. Specifically, we used time-series RNA-seq data from etiolated *Zea mays* (C_4_) and *Oryza sativa* (C_3_) leaf tissues sampled during a de-etiolation process. With this data, we first studied the responses of C_4_ genes in de-etiolated leaves during the greening process; then we used computational approaches to predict potential *cis*-regulatory elements in different segments of major C_4_-related genes; finally, we examined the likelihood of the recruitment of pre-existing *cis*-regulatory elements into C_4_ metabolic genes during C_4_ evolution, and we provide a list of potential recruited *cis*-elements that might serve for further experimental validation.

## Material and methods

### Plant material, RNA isolation, and mRNA sequencing

*Zea mays* ecotype B73 and *Oryza sativa japonica* seeds were sown and cultured in soil in darkness at 28/22 °C on a 16/8h cycle and at 60% humidity for 1 week. The 7-d-old etiolated seedlings were then exposed to continuous light (approx. 200 umol m^–2^ s^–1^ at the surface of the sampled leaves) and illuminated for 24h. Seeds for control experiments were sown and cultured in soil for 1 week with a 16-h (07:00–23:00h) light/8-h night cycle. Leaf sections of about 2cm length were taken from the end third of the leaf (i.e. near the tip), from the third leaf on the plant. Samples from etiolated plants were harvested before the start of illumination (termed 0h, at 09:00h) and then at six other time points into the light period, namely 0.5h, 1h, 3h, 6h, 12h, and 24h. Control samples were harvested at 09:00h. We used six pooled segments for each sample. These samples were immediately frozen in liquid nitrogen and stored at –80 °C until use. Total RNA was extracted employing the TRIzol® protocol and purified with the RNeasy Plant Mini Kit (Qiagen, Hilden, Germany) following the manufacturer’s instructions. The RNA integrity was evaluated by agarose gel electrophoresis and the concentration was checked using a Nanodrop ND-1000 spectrophotometer (Thermo Fisher Scientific, Wilmington, DE, USA). Quality-controlled RNA samples were prepared for sequencing on an Illumina HiSeq 2000 using the Illumina TruSeq^TM^ RNA sample preparation v2 guide (Catalog # RS-122–2001). Library preparation and sequencing were conducted by the Beijing Genomics Institute (Shenzhen, China). Rawreads of Illumina 2000 sequencing data were submitted to the GenBank Short Read Archive (SRA) database (accession number SRX766219).

### Expression profiles and co-expressed genes

The 90-bp pair-end sequencing reads were processed by the FASTX-toolkit pipeline version 0.0.13 (http://hannonlab.cshl.edu/fastx_toolkit/) to remove the adapters. Low-quality reads were then discarded to ensure that more than 70% of the bases in the retained reads possessed a Phred score greater than 30 (indicating a 1‰ sequencing error rate). Read quality was then examined by FastQC (http://www.bioinformatics.babraham.ac.uk/projects/fastqc/). The reads were mapped to the B73 maize genome (http://www.phytozome.net/) with Bowtie 2 version 2.1.0 (http://bowtie-bio.sourceforge.net/bowtie2/index.shtml) (Langmead and Salzberg, 2012) allowing at most three mismatches per read. RPKM values (reads per kilobase of transcript per million mapped reads) were then calculated for each gene. For each gene, the spline function in R (www.r-project.org) was applied to smooth the seven time points.

Regressed curves were then normalized by standard deviation before *k*-means clustering was conducted using R.

### Identification of orthologous gene pairs and C_4_ isoforms

Genome-wide maize and rice orthologous gene pairs were identified by a combination of tools including BBH-LS (Zhang and Leong, 2012), orthoMCL (Chen *et al.*, 2006), Inparanoid (Ostlund *et al.*, 2010), MSOAR2 (Shi *et al.*, 2010), and the Ensembl database (Hubbard *et al.*, 2002). The results were organized based on the following principles. First, the results from BBH-LS, which is the most stringent method (Zhang and Leong, 2012), were used as the basis. Second, if a particular ortholog pair was not reported by BBH-LS but was by other methods, we retained the results of the other methods. Third, if the results from different approaches were in conflict with each other, genes with higher expression values were retained on the assumption that functional genes generally have higher expression. Fourth, only one-to-one orthologous gene pairs were retained. The list of identified orthologous gene pairs is given in Supplementary Table S1 at *JXB* online.

We used the 15 genes related to the C_4_ pathway for detailed analysis. To identify the particular isoform that was recruited into the C_4_ pathway, we first checked whether information on the cell-specificity of its expression was available. If not, the highest-expressed gene in maize among its paralogs was regarded as the C_4_ isoform. Multiple methods including Euclidean distance between expression curves, rank correlation, and mutual information (http://cran.r-project.org/web/packages/infotheo/index.html) were used to measure the level of similarities and differences in the expression patterns between maize genes and their corresponding rice orthologous genes.

### Motif prediction and negative control

To obtain lists of genes that showed similar expression patterns with target C_4_ genes, we applied a *k*-means clustering algorithm to maize and rice genes that showed an average RPKM value greater than 1 (Supplementary Figs S1 and S2). Figure of merit (FOM) values were plotted to choose *k* (Supplementary Fig. S3). In this study, we applied *k*-means clustering with two *k*-values, *k*=80 and *k*=30 (hereafter termed as the *k*80 and *k*30 approaches, respectively) to decrease the false positive rate. Euclidean distances between 3rd-polynomial regressed expression curves of target genes and the rest of the genes falling into the same cluster were calculated, sorted, and the *Z*-score transformed. Genes with a *Z*-score value less than –1.644853 (5% tail) were retained as co-expressed genes for motif prediction. Thus, we were able to classify genes with similar expression patterns into different clusters. For both approaches, five genomic segments of the retained genes – i.e. the 3 kb upstream sequence of the transcription start site (TSS), 5′UTR, 3′UTR, coding sequence (CDS), and intron were extracted with the co-ordinates provided by the phytozome genome annotation (http://www.phytozome.net/). Introns and CDS segments were artificially concatenated by insertion of 10 Ns. Three different methods were combined to predict conserved regulatory motifs: TRAP (Roider *et al.*, 2007) with the aid of TRANSFAC database release 2010.2 (Matys, 2003), Weeder2 (Pavesi *et al.*, 2006; Zambelli *et al*, 2014), and MEME version 4.8.1 (Bailey *et al.*, 2006). The enrichment *P*-value of identified motifs within input sequences compared with the genome background was set to 0.05 for the TRAP, MEME, and Weeder2 outputs. Predicted enriched motifs were mapped to genomic sequences to verify their existences using cisGenome version 2.0 (http://www.biostat.jhsph.edu/~hji/cisgenome) (Ji *et al.*, 2006). During this process, at most two mismatches to the consensus sequence and zero mismatch to the degenerate consensus sequence were allowed (Ji *et al.*, 2006). Alignments between motifs were done using STAMP version 1.1 (Mahony and Benos, 2007) with the maximum *P*-value set to 0.01. Negative controls were used to confirm the reliability of this combined approachby using three different sets of 50 randomly selected genes as the input list to predict motifs. No conserved DNA motifs were identified across the different methods (see Supplementary Table S2).

## Results

### Overview of the effects of illumination on the transcriptome of etiolated maize and rice leaves

In total, about 81.6 and 137.4 million reads were sequenced for maize and rice, respectively. Read counts and number of expressed genes for each time point are listed in Supplementary Table S3. The gross differences of expression patterns between maize and rice leaves during de-etiolation were first assessed by quantifying the transcript abundance in different pathways, as defined by MapMan bincodes (http://mapman.gabipd.org/). This was done by calculating the average RPKM value of all genes involved with a pathway. Comparing maize to rice, we found that the majority of pathways showed similar changes in expression patterns upon illumination. For example, pathways annotated as ‘cell activity’, ‘DNA activity’, ‘hormone metabolism’, and ‘secondary metabolism’ (see Supplementary Table S4) were slightly influenced by light. However, in many other pathways the responses to light exposure differed between maize and rice. For example, genes classified into the ‘cell activity’ pathway did not show much response to illumination in maize, whereas these genes showed slightly decreased expression in rice (Supplementary Table S4). In contrast, genes annotated as ‘C1 metabolism’ showed lower expression in rice in the dark and showed higher expression under illumination, while the expression of these genes was not influenced by illumination in maize (Supplementary Table S4). Genes annotated to be related to the ‘redox’ pathway showed similar expression levels between etiolated and control samples for maize, but their expression was suppressed in the etiolated samples compared with control samples for rice (Supplementary Table S4).

**Fig 1.**
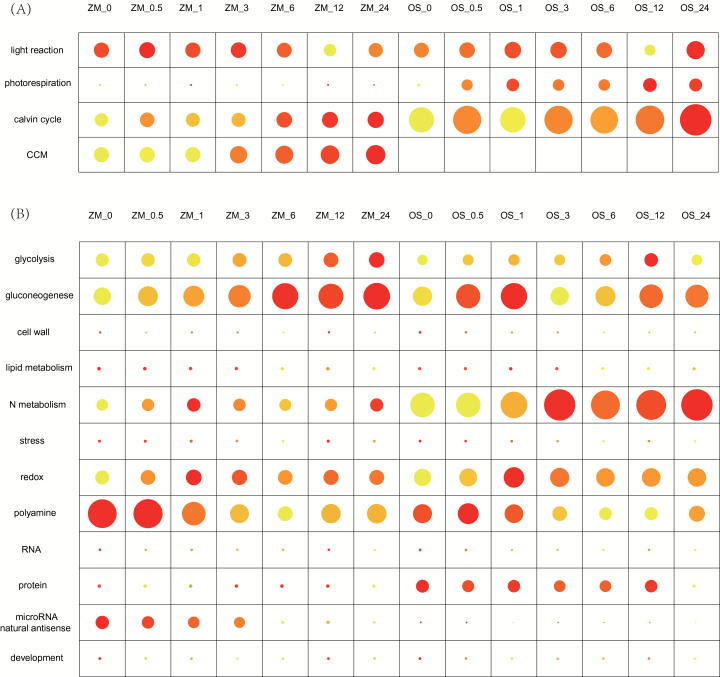
Pathway-level gene expression of maize and rice during the de-etiolation process. The dot size represents the gene expression level across the whole genome. The color code indicates the relative gene expression level within a given pathway, from low (yellow) to high (red). (A) Photosynthetic pathways, and (B) non-photosynthesis related pathways. CCM, CO_2_ concentration mechanism.

Photosynthesis-related pathways were generally up-regulated during greening (Fig. 1A). Genes involved in the light reactions were activated almost immediately in both species, but the expression level peaked much earlier in maize than in rice (Fig. 1A). Photorespiration-related genes showed higher expression in rice than in maize, which is consistent with a higher photorespiratory flux in C_3_ plants than in C_4_ plants. The genes involved in the Calvin–Benson cycle also showed higher expression under illumination in both species, with C_3_ (rice) showing higher expression as compared to C_4_ (maize). Maize showed much higher expression values for genes contributing to the C_4_ CO_2_-concentrating mechanism (CCM) while rice did not show detectable expression of CCM-associated genes. In maize, the expression of genes involved in both the Calvin–Benson cycle and CCM gradually increased and peaked after leaves were illuminated for 24h.

Many pathways other than photosynthesis were also influenced by illumination. For example, ‘micro RNA and natural antisense’ genes showed higher expression in the dark and then decreased upon illumination (Fig. 1B). Similarly, pathways annotated as ‘development’, ‘stress’, ‘RNA activity’, ‘protein activity’, ‘polyamine metabolism’, and ‘tetrapyrrole synthesis’ also showed higher expression in the dark as compared to illumination in both species (Fig. 1B).

### Transcript abundance of C_4_ genes during greening

**Fig 2.**
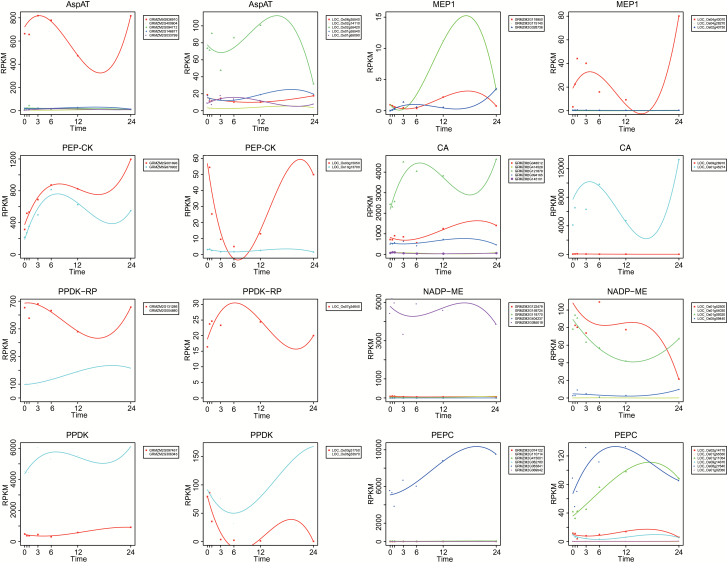
Expression curves of C_4_ gene families. The *x*-axis represents different time points and the *y*-axis represents the RPKM value. Expression curves were 3^rd^-order polynomial regressed, whilst points indicate actual RPKM values.

For the majority of genes involved in the C_4_ pathway in maize and their orthologous genes in rice, only one member of each gene family showed a high level of expression in the mature leaf section during the greening process (Fig. 2). The only exception in maize is *PEP-CK*, i.e. both GRMZM2G001696 and GRMZM5G870932 were detected with high expression, and similar expression patterns were observed (Fig. 2). While we cannot rule out that technical issues related to read mapping could be the cause of this gene expression pattern, it is also possible that either both genes might be involved in the C_4_ pathway in maize or one is playing an important role in housekeeping. In rice, both *NADP-ME* and *PEPC* gene families hold two members with high expression in the leaf during greening (Fig. 2). In addition, these two members in each pair showed different expression patterns. For both *NADP-ME* and *PEPC*, one of the two highly expressed members, i.e., LOC_Os01g52500 for *NADP-ME* and LOC_Os01g11054 for *PEPC*, showed a similar expression pattern to its maize counterpart, while the other member, i.e., LOC_Os01g09320 and LOC_Os08g27840, did not.

**Fig 3.**
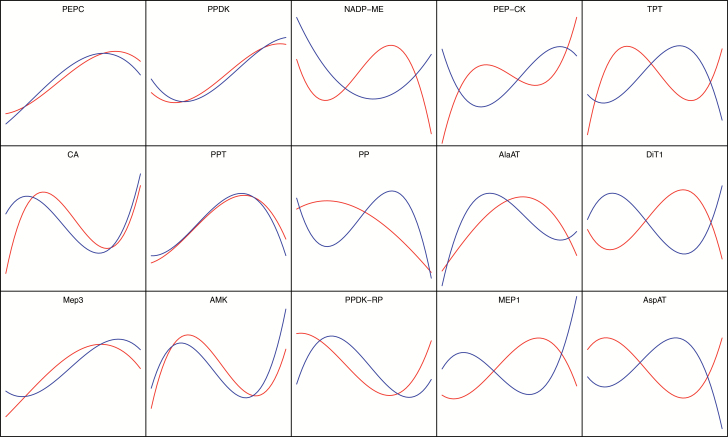
Expression patterns of C_4_ orthologous gene pairs between maize (red) and rice (blue). RPKM values were normalized after 3^rd^-order polynomial regression.

Taking into consideration that C_4_ evolved from the ancestral C_3_ state by recruiting pre-existing components (Sheen, 1999; Hibberd and Covshoff, 2010), we further examined whether these highly expressed genes in the C_4_ gene families were orthologous between maize and rice. We applied a combination of methods including BBH-LS (Zhang and Leong, 2012), orthoMCL (Chen *et al.*, 2006), Inparanoid (Ostlund *et al.*, 2010), MSOAR2 (Shi *et al.*, 2010), and the Ensembl database (Hubbard *et al.*, 2002). When comparing the expression patterns of C_4_ orthologous genes of 15 C_4_ photosynthesis-related genes (Pick *et al.*, 2011) between maize and rice, we categorized the different expression patterns into three different types, namely similar, distinct, and shifted (Fig. 3).

Orthologous genes for *PEPC*, *PPDK*, *PPT*, *Mep3*, and *AMK* showed similar expression patterns between maize and rice if they were normalized to the same scale of expression level (Table 1). However, for all these five genes the maize orthologous genes showed higher expression than their rice counterparts before normalization, in particular for *PPT* and *Mep3*. Although the expression levels of maize *PEPC* and *PPDK* were elevated by approximately two-fold after 24h illumination, we detected relatively high expression of *PEPC* and *PPDK* in maize seedlings under dark conditions, which is in contrast to some previous studies (Sheen and Bogorad, 1987; Langdale *et al.*, 1988).

**Table 1.** Euclidean distances between maize and rice orthologous gene pairs. For columns headed 1–7, the 1st column indicates the pattern observed in Fig. 3, where ‘s’ stands for similar and ‘d’ stands for different; the 2nd column is the Euclid distance between two clusters that maize and rice genes fall into; the 3rd column is the rank correlation coefficient between maize and rice RPKM vectors ordered across time points; the 4th column is the rank correlation coefficient between maize and rice RPKM vectors ordered across genes; the 5th column is the mutual information value calculated by the R package ‘infotheo’ when setting the bin number to be 3; the 6th column is the maximum mutual information value; the 7th column is the random mutual information value by taking the average of 100 permutations of RPKM values across time points

Gene ID	Maize ID	Rice ID	1	2	3	4	5	6	7
*PEPC*	GRMZM2G083841	LOC_Os01g11054	s	2.21	0.82	0.21	0.73	1.00	0.39
*PPDK*	GRMZM2G097457	LOC_Os05g33570	s	0.00	0.54	0.77	0.26	1.00	0.38
*NADP-ME*	GRMZM2G085019	LOC_Os01g09320	d	12.25	0.46	0.31	0.26	1.00	0.38
*PEP-CK*	GRMZM2G001696	LOC_Os03g15050	d	8.26	-0.61	-0.74	0.46	1.00	0.38
*TPT*	GRMZM2G070605	LOC_Os01g13770	d	10.47	-0.11	-0.12	0.26	1.00	0.39
*CA*	GRMZM2G121878	LOC_Os01g45274	s	3.76	0.29	0.03	0.26	1.00	0.39
*PPT*	GRMZM2G174107	LOC_Os08g25624	s	2.14	0.68	0.86	0.46	1.00	0.36
*PP*	GRMZM2G090718	LOC_Os02g52940	d	10.26	0.18	0.28	0.46	1.00	0.40
*AlaAT*	GRMZM2G028379	LOC_Os03g48080	d	8.14	0.64	0.31	0.46	1.00	0.37
*DiT1*	GRMZM2G383088	LOC_Os12g33080	d	13.74	-0.79	-0.81	1.00	1.00	0.39
*Mep3*	GRMZM2G138258	LOC_Os01g72710	s	4.15	0.50	0.59	0.26	1.00	0.40
*AMK*	GRMZM2G178192	LOC_Os08g01770	s	3.54	0.82	0.75	0.73	1.00	0.39
*PPDK-RP*	GRMZM2G131286	LOC_Os07g34640	s	7.46	-0.50	-0.17	0.73	1.00	0.38
*MEP1*	GRMZM2G175140	LOC_Os04g43070	d	13.13	0.25	0.23	0.26	1.00	0.44
*AspAT*	GRMZM5G836910	LOC_Os02g55420	d	12.91	-0.82	-0.42	0.73	1.00	0.39

The majority of the 15 gene pairs, namely *NADP-ME*, *PEP-CK*, *TPT*, *CA*, *PP*, *AlaAT*, *PPDK-RP*, *MEP1*, and *AspAT* showed different expression patterns between maize and rice (Fig. 3), with most of them showing lower expression in the dark in both species (Fig. 3). *CA*, *AlaAT*, and *MEP1* were the only three genes out of the 15 genes for which the expression levels were lower in maize as compared to rice (Fig. 3). *DiT1*, a dicarboxylate transporter that translocates 2-Oxoglutarate (2-OG) or malate across the plastid envelope membrane, showed similar expression patterns but with a ‘phase shift’ between maize and rice (Fig. 3), as reflected by the high mutual information value between the maize and rice transcriptomics data (Table 1).

### Systematic identification of potential regulatory motifs in various genomic regions of C_4_ genes

For each of the 15 C_4_ genes, two gene lists, i.e. derived from either the *k*80 or *k*30 approaches, were used as input for three different methods (TRAP, MEME, and Weeder2) for motif predictions. The predicted short DNA elements for both maize and rice are listed in Supplementary Tables S5 and S6, respectively.

Motifs predicted by the *k*80 and *k*30 approaches covered the majority (179 out of 180 for *k*80 and 180 out of 180 for *k*30) of motifs identified from photosynthesis-enriched gene clusters by Wang *et al.* (2014) (see Supplementary Table S7 and S8), suggesting the validities of the data, routine, and algorithm used in this study. Consistency of the predicted motifs using these two gene lists, i.e. either from the *k*80 or *k*30 results of *k*-means clustering, were checked by motif mapping using STAMP (Table 2, Supplementary Table S9). Overall, about 60% of motifs (for the promoter region, around 85% of the motifs) predicted by the *k*80 approach overlapped with those motifs predicted by the *k*30 approach (Table 2, Supplementary Table S9), which represents about 36% of the total number of motifs predicted by the *k*30 approach (Supplementary Tables S5 and S6). To make the predictions more reliable, we based our further analysis on the overlapped motifs predicted by both the *k*80 and *k*30 approaches.

**Table 2.** Mapping motifs predicted by the *k*80 and *k*30 approaches. Total predicted motifs is the total number of motifs predicted by the gene list obtained by *k*-mean clustering using the *k*80 approach. Mapped motifs is the number that could be mapped to motifs predicted by the *k*30 approach by STAMP with a *P*-value cut-off set at 0.01

Genomic section	Total predicted motifs	Mapped motifs	Mapped rate
Maize promoter	747	645	86.3%
Rice promoter	545	461	84.6%
Maize 5UTR	311	175	56.3%
Rice 5UTR	208	101	48.6%
Maize 3UTR	385	217	56.4%
Rice 3UTR	311	217	69.8%
Maize CDS	531	382	71.9%
Rice CDS	440	333	75.7%
Maize intron	538	436	81.0%
Rice intron	556	184	33.1%

**Fig 4.**
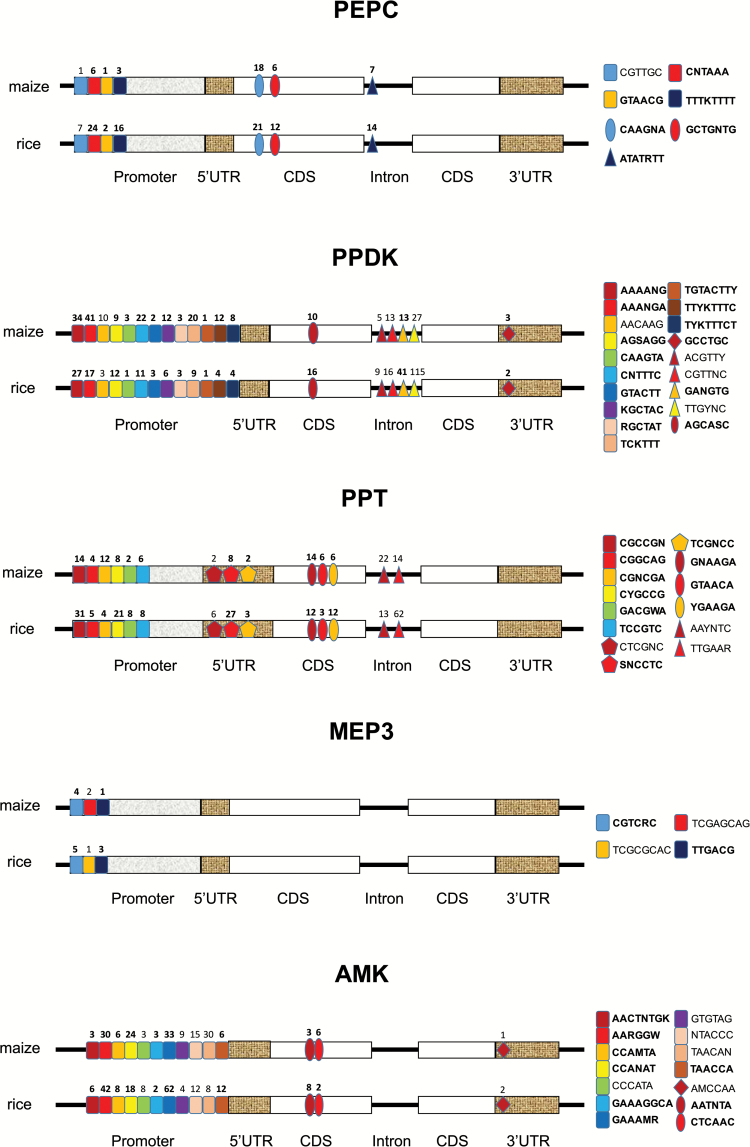
Diagram showing numbers and species of conserved DNA motifs between maize and rice. Conserved DNA motifs identified with the *k*80 appraoch are marked with color as indicated in the keys, and the number of mapped sites are shown. Overlapping results between the *k*80 and *k*30 approaches are marked in bold in the keys.

Motifs identified for maize C_4_ genes were aligned against motifs identified for rice orthologous genes (see Supplementary Table S10). Analysis of the identified motifs showed that except for maize *AMK* 5′UTR, maize *PP* 3′UTR, maize *MEP1* introns, rice *MEP1* introns, and *DiT1* 5′UTR, all other segments of C_4_-related genes contain conserved motifs between maize and rice (Tables 3 and 4, Supplementary Table S10, and Fig. 4). Interestingly, although no *cis*-regulatory motifs have been validated experimentally, a large number of conserved motifs were identified in intron regions (Supplementary Table S10), further indicating that introns might be involved with active regulation in agreement with previous findings (Chang and Sun, 2002; Le Hir *et al.*, 2003). The motifs predicted for C_4_ genes conserved between maize and rice are shown in Table 3. Diagrams showing the relative number of motifs in different genomic segments are shown in Fig 4. The identified motifs conserved between C_3_ and C_4_ orthologs might be related to photosynthesis or to the morphogenesis during the de-etiolation process in general.

**Table 3.** Likelihood of identifying *cis* elements in genomic regions of C_4_ orthologous genes. ‘√’ indicates that conserved motifs were identified between the different methods; ‘X’ indicates that no conserved motifs were identified The numbered columns are as follows: 1, *PEPC*; 2, *PPDK*; 3, *NADP-ME*; 4, *PEP-CK*; 5, *TPT*; 6, *CA*; 7, *PPT*; 8, *PP*; 9, *AlaAT*; 10, *DiT1*; 11, *Mep3*; 12, *AMK*; 13, *PPCK-RP*; 14, *MEP1*; 15, *AspAT*.

	Section	1	2	3	4	5	6	7	8	9	10	11	12	13	14	15
Maize	Promoter	√	√	√	√	√	√	√	X	√	√	√	√	√	√	√
	5′UTR	√	X	√	√	√	√	X	√	√	√	X	X	X	√	√
	3′UTR	√	√	√	X	√	√	X	√	X	√	√	√	√	√	√
	Intron	√	X	√	X	√	√	√	√	√	√	X	X	√	√	√
	CDS	√	√	√	X	√	√	√	X	√	√	√	√	√	√	√
Rice	Promoter	√	√	√	√	√	√	√	√	√	√	√	X	√	√	√
	5′UTR	√	√	√	X	√	X	√	√	√	X	√	X	√	X	√
	3′UTR	√	√	√	√	√	√	√	X	√	√	√	√	√	√	X
	Intron	√	√	X	X	√	√	√	X	√	X	√	X	√	X	√
	CDS	√	√	√	√	√	√	√	√	√	√	√	X	√	√	√

**Fig 5.**
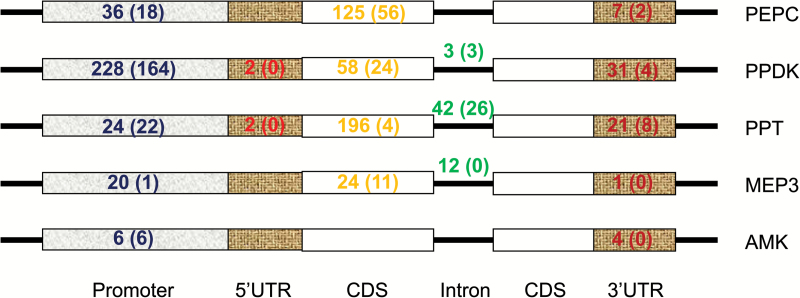
The number of recruited motif sites in different segments of C_4_ genes. The total number of mapped sites for potential recruited motifs in maize identified using the *k*80 approach are given in the corresponding genomic segments, and the number of overlapping motifs is indicated in brackets.

Furthermore, we also identified those motifs that might be recruited into C_4_ metabolic genes from genes unrelated to C_4_ photosynthesis. These motifs were selected based on the following criteria: (1) this particular short DNA motif is enriched in both a C_4_ gene and a non-C_4_ gene that show similar expression patterns with the target C_4_ gene; and (2) this particular short DNA motif exists in a maize C_4_ gene but not in its rice C_4_ orthologous genes. The first criterion ensures that this chosen motif might be associated with the regulation of this particular expression pattern of a C_4_ gene. The second criterion ensures that this particular motif does not exist in the C_3_ orthologous gene. The potential recruited *cis*-regulatory motifs are listed in Supplementary Table S11 and their distribution in different genomic segments is shown in Fig. 5.

**Table 4.** The most conserved motifs predicted for maize and rice. The motifs listed in this table satisfy the following criteria: (1) conserved between at least two prediction methods; (2) conserved between maize and rice orthologous genes; and (3) conserved across maize or rice genes. ‘–’ indicates that no motifs were identified under the specified conditions. Overlapping results between the *k*80 and *k*30 approaches are indicated in bold. Numbers in brackets indicate the numbers of copies of this particular motif in the corresponding maize and rice genomic segments, respectively. M, A or C; R, A or G; W, A or T; S, C or G; Y, C or T; K, G or T; V, not T; H, not G; D, not C; B, not A

Section	*PEPC*	*PPDK*	*PPT*	*Mep3*	*AMK*
**Promoter**	CGTTGC (1,7)	**AAAANG (34,27**)	**CGCCGN (14,31**)	**CGTCRC (4,5**)	**AACTNTGK (3,6**)
	**CNTAAA (6,24**)	**AAANGA (41,17**)	**CGGCAG (4,5**)	TCGAGCAG (2,0)	**AARGGW (30,42**)
	**GTAACG (1,2**)	AACAAG (10,3)	**CGNCGA (12,4**)	TCGCGCAC (0,1)	**CCAMTA (6,8**)
	**TTTKTTTT (3,16**)	**AGSAGG (9,12**)	**CYGCCG (8,21**)	**TTGACG (1,3**)	**CCANAT (24,18**)
		**CAAGTA (3,1**)	**GACGWA (2,8**)		CCCATA (3,8)
		**CNTTTC (22,11**)	**TCCGTC (6,8**)		**GAAAGGCA (3,2**)
		**GTACTT (2,3**)			**GAAAMR (33,62**)
		**KGCTAC (12,6**)			GTGTAG (9,4)
		**RGCTAT (3,3**)			NTACCC (15,12)
		**TCKTTT (20,9**)			TAACAN (30,8)
		**TGTACTTY (1,1**)			**TAACCA (6,12**)
		**TTYKTTTC (12,4**)			
		**TYKTTTCT (8,4**)			
**5′UTR**	–	–	CTCGNC (2,6)	–	–
			**SNCCTC (8,27**)		
			**TCGNCC (2,3**)		
**3′UTR**	–	**GCCTGC (3,2**)	–	–	AMCCAA (1,2)
**Intron**	**ATATRTT (7,14**)	ACGTTY (5,9)	AAYNTC (22,113)	–	–
		CGTTNC (13,16)	TTGAAR (14,62)		
		**GANGTG (13,41**)			
		TTGYNC (27,115)			
**CDS**	**CAAGNA (18,21**)	**AGCASC (10,16**)	**GNAAGA (14,12**)	-	**AATNTA (3,8**)
	**GCTGNTG (6,12**)		**GTAACA (6,3**)		**CTCAAC (6,2**)
			**YGAAGA (6,12**)		

We also found conservation between the predicted motifs in this study and the motifs identified by previous experimental approaches (Sheen, 1999, Gowik *et al.*, 2004; Supplementary Table S12). In addition, to facilitate the identification of potential transcription factors that might bind to these candidate motifs, we have further listed the motifs with information for binding transcription factors available from the PLACE database (Higo *et al.*, 1999) in Supplementary Table S13.

## Discussion

This study reports the changes in the transcriptomics of etiolated leaves of maize and rice upon illumination and also systematically predicts potential *cis*-regulatory motifs in C_4_-related genes. We provide evidence for potential recruitment of *cis*-regulatory motifs from non-photosynthesis genes into C_4_ metabolic genes during evolution of C_4_ photosynthesis. In this section, we first discuss the rationale of using the de-etiolation process as a model system for studying C_4_ photosynthesis. Next, we discuss a number of differences in the transcriptome responses during the de-etiolation process between maize and rice. Finally, we discuss the evidence from this study supporting potential recruitment of pre-existing regulatory elements from non-photosynthetic genes into C_4_ photosynthesis.

### The de-etiolation system as a model to study regulation of C_4_ photosynthesis

We chose the greening process of etiolated leaves for this study for the following reasons. First, light serves both as the energy source for photosynthesis and as an environmental signal during photosynthesis development (Deng and Quail, 1999), and many photosynthetic genes show altered expression levels during light induction (Shen *et al.*, 2009) together with changes of many other genes, which makes it possible to conduct clustering analysis and to perform motif identification based on genes in the same cluster. Second, with multiple sampling times throughout the de-etiolation process, time-series expression patterns for each gene can be established and used for inferring regulator–target gene pairs. With maize being a C_4_ species and rice being a C_3_ species, comparison of the motifs over-enriched in the C_4_ target genes and the associated genes in the same cluster, but not in the C_3_ orthologous genes, provides an opportunity to identify motifs that were potentially recruited during C_4_ evolution. Using expression data based on the de-etiolation process is not a new idea. In fact, this system was one of the most widely used to identify *cis*-elements controlling individual enzymes (Schaffner and Sheen, 1991; Sheen, 1991; Kausch *et al.*, 2001). Recently, this system has also been used to demonstrate that during C_4_ evolution the histone modification code was recurrently recruited into different lineages of C_4_ lineages (Heimann *et al.*, 2013).

### Transcriptomic responses of maize and rice during the de-etiolation process

Alhough the de-etiolation process has been reported extensively in the literature (Schaffner and Sheen, 1991; Sheen, 1991; Kausch *et al.*, 2001), so far there has been no comparative transcriptomic data for maize and rice during the de-etiolation process. A number of distinguishing features in the transcriptomes between maize and rice were identified here. Firstly, as expected, we observed decreased expression of genes involved in photorespiration and increased expression of genes involved in the CO_2_-concentrating mechanism (CCM) in the C_4_ plant, consistent with earlier reports for other C_4_ species (Bräutigam *et al.*, 2011, 2014; Gowik *et al.*, 2011) (Fig. 1A). The expression of enzymes in the Calvin–Benson cycle showed a slightly lower level in maize compared to rice. The decreased expression of RuBisCO reflects a decreased demand for this enzyme under elevated CO_2_ levels in bundle sheath cells. In addition, we found that expression of genes involved in other Calvin–Benson cycle enzymes, such as glyceraldehyde-3-phophate dehydrogenase (*GAPDH*), RuBisCO activase (*RCA*), and fructose-bisphosphate aldolase, also showed decreased levels. We also observed decreased expression of enzymes involved in nitrogen metabolism and protein synthesis (Fig. 1B), which have also been observed in earlier comparative studies of C_3_ and C_4_ transcriptomics (Bräutigam *et al.* 2011, 2014; Gowik *et al.* 2011). This decrease might be related to the decreased content of RuBisCO, one of the most nitrogen-costly proteins in the leaf (Ellis, 1979; Dhingra *et al.*, 2004), and hence a decreased demand for protein synthesis (Piques *et al.*, 2009).

Nearly all genes involved in photosynthesis showed up-regulation during the de-etiolation process (Fig. 1A) (Bradbeer, 1969; Kobayashi *et al.*, 1980; Shen *et al.*, 2009). Interestingly, although genes encoding components of the photosynthetic light reaction in both rice and maize were up-regulated upon exposure to light, the enzymes involved in the light reactions showed faster responses to light in maize compared to rice, i.e. they reached their peak expression levels faster than in rice (Fig. 1A). In contrast, the response speeds of the Calvin–Benson cycle enzymes in maize were similar to C_3_ leaves (Fig. 1A). This might indicate that the expression patterns of genes in the light reaction may be an essential step before light-induced establishment of cell-specific accumulations of Calvin–Benson cycle enzymes, as suggested by an earlier study (Langdale *et al.*, 1988).

Different genes involved in the C_4_ cycle showed distinct expression patterns between rice and maize. Five C_4_ photosynthesis-related enzymes, *PEPC*, *PPDK*, *PPT*, *Mep3*, and *AMK*, showed similar expression patterns between maize and rice (Fig. 3), suggesting potentially conserved regulatory mechanisms for these genes between the two species. *DiT1*, aspartate aminotransferase (AspAT), MEP1, triose phosphate transporter (TPT), and PEP carboxykinase (PEP-CK) showed shifted expression patterns between maize and rice. *DiT1*, *AspAT*, *MEP1*, and *PEP-CK* are key enzymes related to the operation of the C_4_ pathway in mature maize leaves (Pick *et al.*, 2011). *DiT1* has been reported as a crucial protein at the interface between carbon and nitrogen metabolism (Schneidereit *et al.*, 2006). In addition, *PEP-CK* and *AspAT* also play an important role in the interaction of carbon and nitrogen metabolism (Walker *et al.*, 1999). Considering that both maize and rice N metabolism and gluconeogenesis pathways showed a strong circadian rhythm (Fig. 1B), it is possible that some of the regulatory mechanisms of the current C_4_ pathway might have recruited pre-existing mechanisms from the circadian rhythms.

### *Cis*-regulatory motifs related to C_4_ photosynthesis

As a common theme of evolution, C_4_ evolved from C_3_ photosynthesis by recruiting pre-existing elements. For example, all C_4_ metabolic enzymes exist in C_3_ plants and play important house-keeping roles in their C_3_ host (Aubry *et al.*, 2011). The increased bundle sheath size and chloroplast number in bundle sheath cells in some plants growing in arid regions might represent an adaptation strategy to cope with drought stress (Griffiths *et al.*, 2013). Recent evidence has suggested that even the regulatory elements, such as the 240-nt element in the coding sequence of *NAD-ME* and the regulatory elements in the UTR region of *CA* and *PPDK*, also pre-exist in C_3_ ancestor enzymes as well (Brown *et al.*, 2011; Kajala *et al.*, 2012). This raises an intriguing hypothesis that recruitment of pre-existing *cis*-motifs might have been a common mechanism for evolution of regulatory elements during C_4_ emergence. This study provides new evidence supporting this hypothesis.

We systematically identified potential *cis*-regulatory motifs that might be involved in regulating C_4_ photosynthesis genes. Given that earlier reports have shown that promoter regions (Gowik *et al.*, 2004), coding sequence (Brown *et al.*, 2011), 5′UTR (Marshall *et al.* 1997; Patel *et al.* 2004, 2006), 3′UTR (Ali and Taylor, 2001; Lai *et al.*, 2002; Kajala *et al.*, 2012), and intron regions can harbor *cis*-regulatory motifs controlling cell-specific expression, we examined all these genomic regions. Consistent with previous reports, potential candidate motifs were identified in all these different regions of genes (Tables 3 and 4, Figs 4 and 5). Nearly all the motifs identified previously through experimental approaches in maize (Sheen, 1999) were also identified in our predictions (see Supplementary Table S12). However, motifs identified earlier in dicots (e.g. Gowik *et al.*, 2004; Williams *et al.*, 2016) were not identified in this analysis, possibly due to different regulatory mechanisms controlling expression of C_4_ genes between monocots and dicots.

The distributions of the identified *cis*-motifs show some distinct features. First, a *cis* motif can reside in more than one segment of a gene. For example, the Dof1 binding motif AAAAGG was predicted to reside in both the promoter region and the intron region of *PEPC* (see Supplementary Table S12). Second, a *cis*-regulatory motif may regulate more than one gene. For example, the Dof1 binding motif AAAAGG was predicted to exist in 5′ flanking sequence of *PEPC* (as reported by Sheen, 1999), in the maize *AlaAT* promoter sequences (Supplementary Table S12), and in the intron regions of maize *PEPC* and *NADP-ME* (Supplementary Table S12), suggesting reuse of the same motif in regulating multiple C_4_ genes, a phenomenon shown earlier in C_4_ genes in dicots (Williams *et al.*, 2016).

With the identified *cis*-regulatory motifs, we examined whether it is possible for a *cis*-regulatory motif to be recruited from a gene unrelated to C_4_ photosynthesis to a C_4_ gene. To do this, we identified motifs that exist in maize C_4_ genes and genes showing the same expression patterns but not in their rice orthologous genes. Considering that these motifs were identified based on sequence information for genes in the same cluster, these motifs might have been potentially recruited into C_4_ metabolic genes from those genes unrelated to them (Supplementary Tables S10 and S11, Fig. 5). These data suggest that genes sharing similar expression patterns with C_4_ metabolic genes might have been a rich source of *cis*-regulatory elements recruited into C_4_ genes during the evolution of C_4_ photosynthesis. Indeed, many genes in the bundle sheath cells of C_3_ plants showed highly specialized cell-specific expression and play particular metabolic roles, e.g. the bundle sheath cells of *Arabidopsis thaliana* show a strong preference for sulfur and glucosinolate metabolism (Aubry *et al.*, 2014). Therefore, the required regulatory metabolism for establishing cell specificity, including both the *cis*-element and the *trans*-factors, is in place in C_3_ plants. It should be much easier to recruit them into genes encoding metabolic enzymes related to C_4_ photosynthesis, as compared to evolving *de novo* mechanisms for conferring cell specificity. Our recent anlaysis showed that transposons might have participated in such processes to recruit motifs in the promoter regions (Cao *et al.*, 2016). Here, we show that the potentially recruited motifs reside in all regions of C_4_ genes (Fig. 5). In fact, many of our potentially recruited motifs overlap with bundle-sheath cell-specific motifs identified by Wang *et al.* (2014) (see Supplementary Tables S7 and S8). However, it is worth pointing out a caveat that the potentially recruited motifs identified here could perhaps represent differences between BEP clade and panicoid grasses (Brutnell *et al.*, 2010), rather than differences between C_3_ and C_4_ photosynthesis. Detailed functional and evolutionary studies of these identified *cis*-motifs are now needed to clarify their significance to C_4_ gene expression, in particular to establish their cell-specific expression patterns.

### False positive discovery rate and negative control for *cis*-element identification

Computational identification of *cis*-elements has the caveat of having a high false-positive rate. In this study, we took a number of measures to overcome this shortcoming of computational approaches, as follows.

(1) Combining multiple approaches for *cis*-element identification. In this study we applied three approaches, namely TRAP, MEME, and Weeder, to predict enriched short DNA motifs. Candidate motifs were retained only if they were conserved between at least two methods. (2) Conservation between species. Given that genes with similar expression patterns are likely to share common regulatory mechanisms, we have predicted enriched motifs for maize and rice orthologous genes, which were then used to detect the shared motifs between *PEPC*, *PPDK*, *PPT*, *Mep3*, and *AMK*. (3) Conservation between different genes. Given that almost all C_4_ genes showed higher expression in maize compared to rice, it is likely that they might share some conserved regulatory mechanisms to achieve this higher gene expression. Thus, we required identified short DNA motifs to exist in at least two C_4_ genes to increase their likelihood to be real functional *cis*-elements. (4) Appearance frequencies. Given that motifs with multiple occurrences are more likely to act as binding sites of transcription factors, we checked the appearance frequencies of the identified cis-regulatory motifs. (5) We also conducted a negative control and the results showed that although some motifs could be identified by randomly selected sequences, no results were retained after a conservation check across different methods (see Supplementary Table S2). (6) Finally, when we used the *k*-means clustering approach to identify the genes in the same cluster, we used two different cluster numbers. Only motifs identified by analysis with both clustering numbers were retained in this study in order to decrease the false-positive rate. Although these stringent measures will effectively decrease the false-positive rates for mis-identifying new motifs, they might also lead to the possible loss of potential cis-regulatory motifs. Therefore, the reported motifs in this study might represent only a conserved list of the potential motifs in these C_4_ genes.

## Conclusions

Studying the molecular mechanisms controlling C_4_ genes is a major focus of current C_4_ photosynthesis research. This study systematically characterized the expression patterns of photosynthesis genes during the de-etilation process, and further identified *cis*-regulatory motifs potentially related to C_4_ photosynthesis genes. Although most of the C_4_ photosynthesis genes showed similar expression patterns between maize and rice, many C_4_ photosynthesis genes, in particular *DiT1*, aspartate aminotransferase, *PEP-CK*, and triose phosphate transporter, showed shifted expression patterns, suggesting a possible recruitment of pre-existing regulatory mechanisms controlling the circadian rhythm during C_4_ emergence. During the process of identifying *cis*-regulatory elements, we took several measures to decrease the potential false-positive rate by using a number of motif prediction methods and using more than one clustering number in the *k*-means clustering. Our analysis shows the widespread existence of *cis*-motifs in different segments of C_4_ genes. Finally, considering that many motifs reside in C_4_ genes and in genes showing similar expression patterns to C_4_ genes in maize while they do not reside in their C_3_ orthologs in rice, we suggest the possibility of recruitment of such motifs from genes other than photosynthesis genes into C_4_ photosynthesis genes.

## Supplementary Data

Supplementary data are available at *JXB* online.

Figure S1. Eighty clusters of maize and rice expressed genes, with the expression curve of each member of a cluster plotted together with the average value of all genes falling into the same cluster.

Figure S2. Thirty clusters of maize and rice expressed genes, with the expression curve of each member of a cluster plotted together with the average value of all genes falling into the same cluster.

Figure S3. Figure of merits of randomly selected genes.

Table S1. One-to-one orthologous gene pairs used in this study identified by a combination of methods.

Table S2. Negative control for cross-methods prediction of DNA motifs.

Table S3. Statistics for RNA-seq samples.

Table S4. Average expression levels of MapMan pathways for maize and rice.

Table S5. Identified motifs for each genomic section of 15 C_4_ gene pairs using the gene list obtained by *k*80 of the *k*-means clustering approach.

Table S6. Identified motifs for each genomic section of 15 C_4_ gene pairs using the gene list obtained by *k*30 of the *k*-means clustering approach.

Table S7. Comparison of identified motifs between the *k*80 approach and the leaf gradient data in Wang *et al.* (2014).

Table S8. Comparison of identified motifs between the *k*30 approach and the leaf gradient data in Wang *et al.* (2014).

Table S9. Comparison of identified motifs between the *k*80 and *k*30 approaches.

Table S10. Comparison of identified motifs between maize and rice C_4_ orthologous genes for the overlapping results between the *k*80 and *k*30 approaches.

Table S11. Potentially recruited motifs and their number of matching sites in corresponding genomic segments.

Table S12. Comparison of identified overlapping motifs between the *k*80 and *k*30 approaches with the motifs summarized in Sheen (1999).

Table S13. Comparison of identified motifs with the PLACE database.

Supplementary Data
