# Systems analysis of *cis*-regulatory motifs in C_4_ photosynthesis genes using maize and rice leaf transcriptomics data during a process of de-etiolation

**DOI:** 10.1093/jxb/erw461

**Published:** 2016-12-22

**Authors:** Jiajia Xu, Andrea Bräutigam, Yuanyuan Li, Andreas P. M. Weber, Xin-Guang Zhu

**Affiliations:** 1 CAS Key Laboratory of Computational Biology and State Key Laboratory for Hybrid Rice, CAS-MPG Partner Institute for Computational Biology, Shanghai Institutes for Biological Sciences, Chinese Academy of Sciences, Shanghai 200031, China; 2 Institute of Plant Biochemistry, Cluster of Excellence on Plant Sciences (CEPLAS), Heinrich-Heine University, 40225 Düsseldorf, Germany; 3 Current address: Institute of Developmental and Molecular Biology of Plants, Plant Molecular Physiology and Biotechnology Group, Heinrich Heine University, 40225 Düsseldorf, Germany

*Journal of Experimental Botany.* (2016) 67(17): 5105–5117. doi:10.1093/jxb/erw275

The authors of the above paper would like to acknowledge Yuanyuan Li as co-author for her contribution on the raw RNA-SEQ data used for the bioinformatics analyses presented in the paper. The authors of the paper are therefore as follows:

Jiajia Xu^1^, Andrea Bräutigam^2^, Yuanyuan Li^1,3^, Andreas P. M. Weber^2^, Xin-Guang Zhu^1,*^

^3^ Current address: Institute of Developmental and Molecular Biology of Plants, Plant Molecular Physiology and Biotechnology Group, Heinrich Heine University, 40225 Düsseldorf, Germany

This has been corrected above and in the article.

