# Supplementary material for: Systems analysis of cis-regulatory motifs in C4 photosynthesis genes using maize and rice leaf transcriptomic data during a process of de-etiolation
Source: J Exp Bot. 2016 Jul 19;67(17):5105–17. doi: 10.1093/jxb/erw275 (PMC5014158; doi:10.1093/jxb/erw275)
Supplement: Supplementary Data [file supp_67_17_5105__index.html]

Systems analysis of cis-regulatory motifs in C4 photosynthesis genes using maize and rice leaf transcriptomic data during a process of de-etiolation — Systems analysis of cis-regulatory motifs in C4 photosynthesis genes using maize and rice leaf transcriptomic data during a process of de-etiolation — Supplementary Data 

# Systems analysis of *cis*-regulatory motifs in C4 photosynthesis genes using maize and rice leaf transcriptomic data during a process of de-etiolation

## Supplementary Data

Data files

- supplementary\_figures\_S1\_S3.pdf - Supplementary Data
- supplementary\_table\_S1.xlsx - Supplementary Data
- supplementary\_table\_S2.xlsx - Supplementary Data
- supplementary\_table\_S3.xlsx - Supplementary Data
- supplementary\_table\_S4.xlsx - Supplementary Data
- supplementary\_table\_S5.xlsx - Supplementary Data
- supplementary\_table\_S6.xlsx - Supplementary Data
- supplementary\_table\_S7.xlsx - Supplementary Data
- supplementary\_table\_S8.xlsx - Supplementary Data
- supplementary\_table\_S9.xlsx - Supplementary Data
- supplementary\_table\_S10.xlsx - Supplementary Data
- supplementary\_table\_S11.xlsx - Supplementary Data
- supplementary\_table\_S12.xlsx - Supplementary Data
- supplementary\_table\_S13.xlsx - Supplementary Data
