# Supplementary material for: Systems analysis of cis-regulatory motifs in C4 photosynthesis genes using maize and rice leaf transcriptomic data during a process of de-etiolation
Source: J Exp Bot. 2016 Jul 19;67(17):5105–17. doi: 10.1093/jxb/erw275 (PMC5014158; doi:10.1093/jxb/erw275)

Figure S1. Eighty clusters of maize and rice expressed genes. RPKM values were polynomial regressed and then normalized to the same scale. The expression curve of each member of a cluster is plotted in grey and the average value of all genes falling into the same cluster is plotted in red.

Supplementary Figure 1

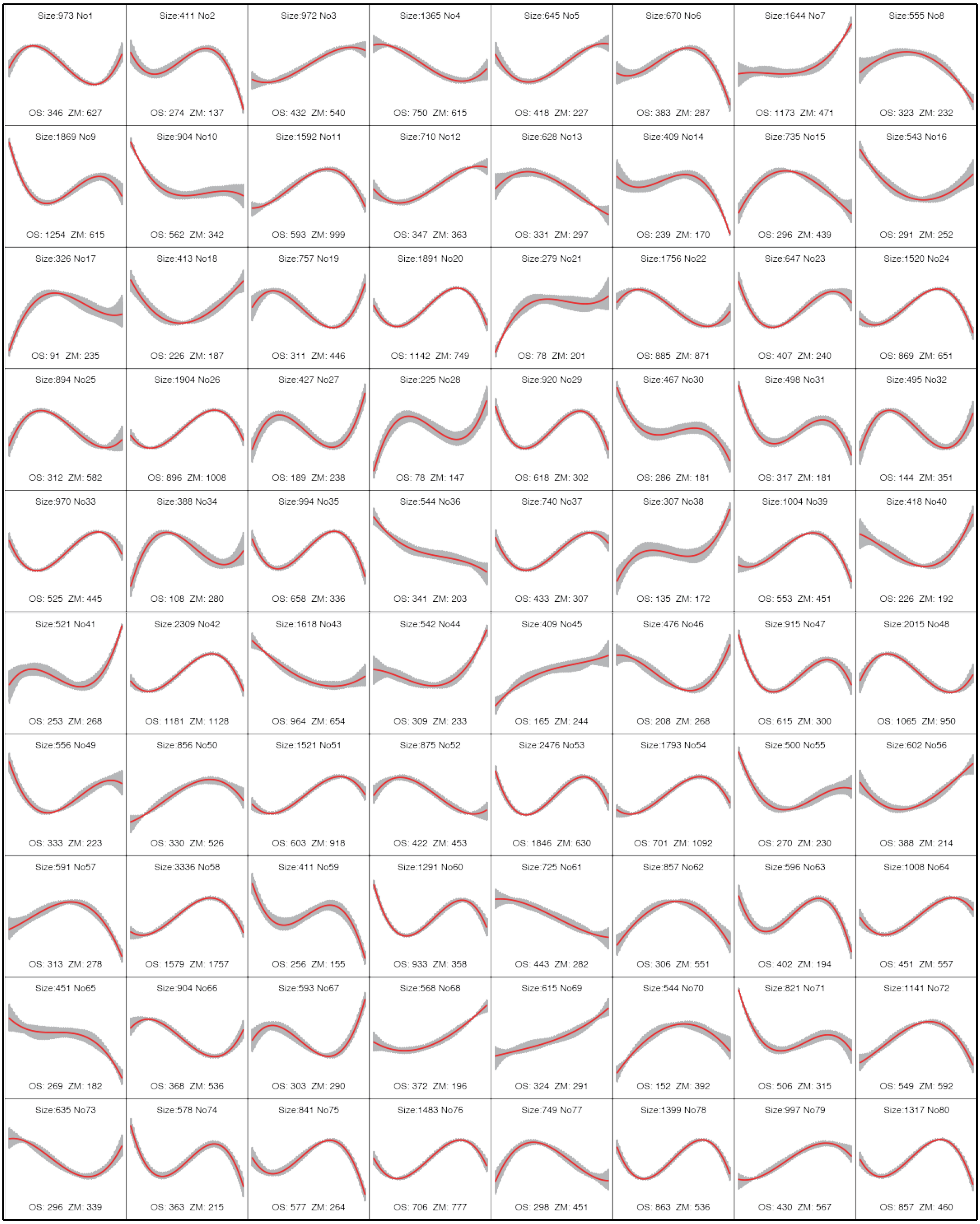

Figure S2. Thirty clusters of maize and rice expressed genes. RPKM values were polynomial regressed and then normalized to the same scale. The expression curve of each member of a cluster is plotted in grey and the average value of all genes falling into the same cluster is plotted in red.

Supplementary Figure 2

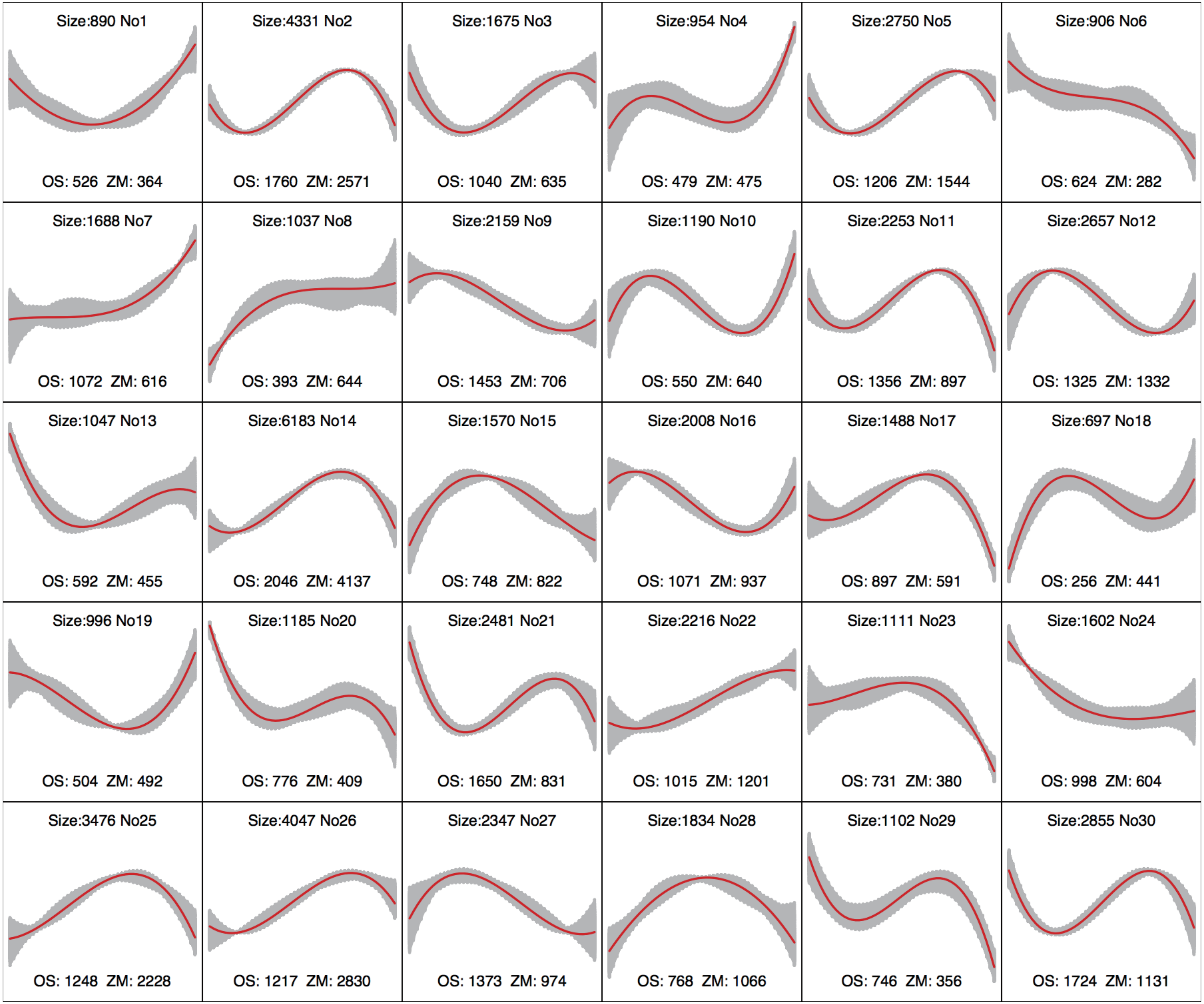

Figure S3. Figure of merits of randomly selected genes. The *x*-axis represents the number of clusters and the *y*-axis represents the FOM value calculated by the R package clValid (Brock *et al*. 2008).

Brock G, Pihur V, Datta S, Datta S. 2008. clValid : an R package for cluster validation. Journal of Statistical Software 25, 1–22.

Supplementary Figure 3

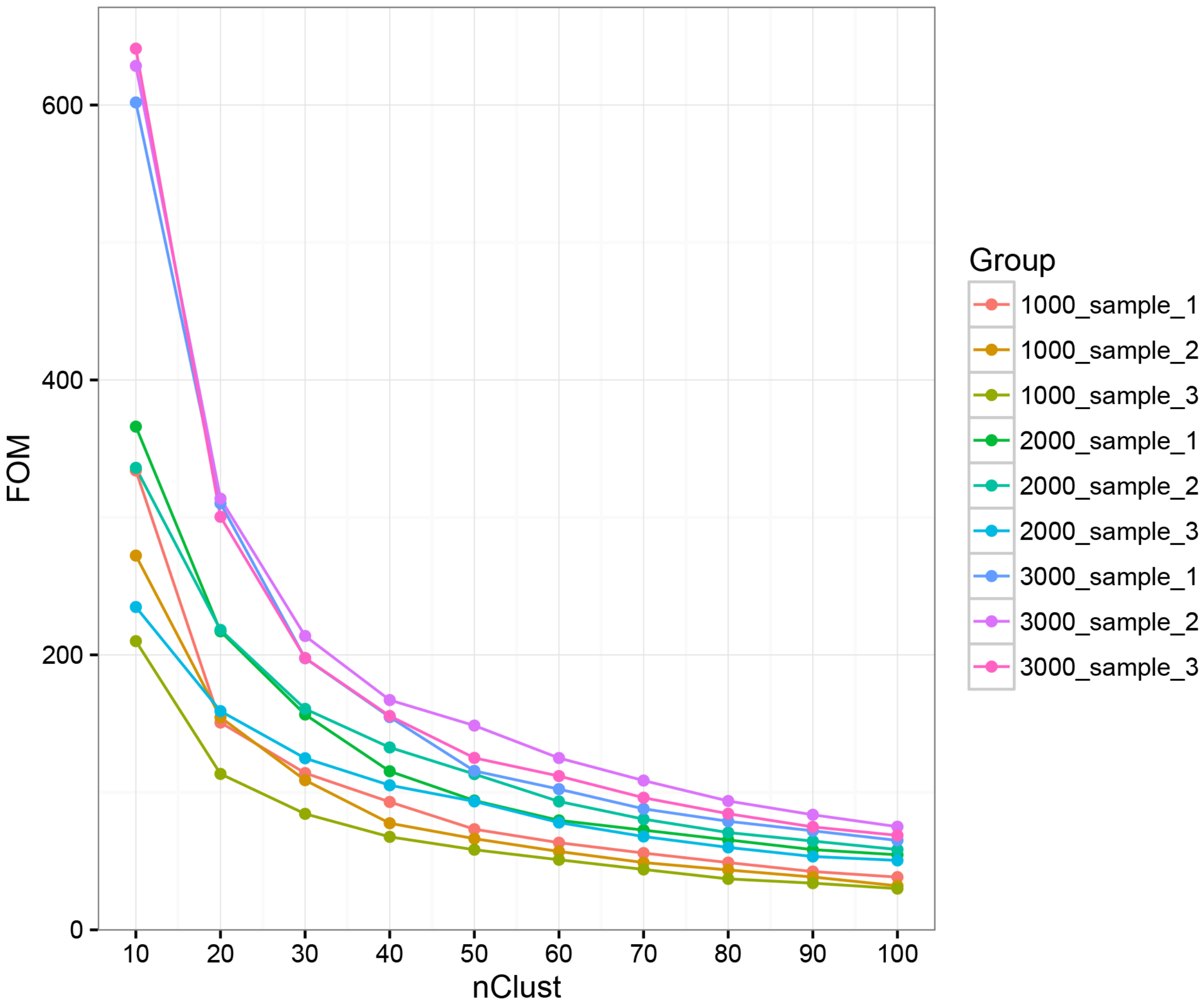

Supplement: Supplementary Data [file supp_erw275_supplementary_figures_S1_S3.pdf]
